# An Analysis of Young Clients' Communications About Their Suicidality on a Text Message Helpline: “I'm Scared of What I Might Do to Myself”

**DOI:** 10.3389/fpsyt.2022.925830

**Published:** 2022-07-14

**Authors:** Jeanne Van Wyk, Kerry Gibson

**Affiliations:** School of Psychology, The University of Auckland, Auckland, New Zealand

**Keywords:** youth suicide, crisis helpline, youth mental health, suicide prevention, text counseling

## Abstract

**Background:**

Youth suicide is a major international concern and prevention is a priority. In most cases suicidal behavior would be preceded by a period of suicidal ideation. Although feeling suicidal is recognized as a risk factor for suicide, there is little research which captures young people's own experience of suicidality in a moment of crisis.

**Aims:**

This study aimed to explore young people's own accounts of their suicidality in the moment in which they experienced it.

**Method:**

This qualitative study examined clients' experience of suicidality as communicated during a text message helpline counseling interaction. The data consisted of 125 text transcripts of an interaction during which a client was experiencing suicidality. These were obtained from a New Zealand based youth helpline service. The data was analyzed using thematic analysis.

**Findings:**

The analysis showed that clients' experienced suicidality as a normal part of their life; that it was understood as a form of coping and that it was seen as a legitimate way to communicate distress. Clients described rapid fluctuations in the intensity of their suicidality and a feeling of being out of control. Despite this, they also communicated ambivalence about acting on their suicidality, and a recognition of the need to get help.

**Conclusions:**

This study offered unique insights into young people's experience of suicidality and opens up opportunities for prevention. It underlines the importance of identifying chronic suicidality early and providing intervention and support prior to a suicidal crisis. The findings point to the potential that text counseling services might have in providing support to young people who are experiencing suicidality in the moment that they need this.

## Introduction

Youth suicide is a major global health concern (1, 2), and youth suicide rates in New Zealand are the second highest in the developed world (3). There are, however, significant challenges in providing help to young people who are experiencing suicidality (4, 5). Some of these challenges might be attributed to young people's reluctance to reach out for help (6–8). However, research also indicates that services may be failing to provide timely and helpful support to young people experiencing suicidality (9). It is vital to improve our knowledge of how young people experience suicidality to identify risks and opportunities to prevent suicide in this vulnerable age group.

While not all young people who experience suicidal ideation will go on to attempt suicide, this is thought to be a necessary precursor to suicide attempts (10), with some research suggesting that suicidal ideation predicts future suicide attempts (11, 12). Suicidal ideation is thought to occur relatively frequently amongst young people, with some estimates as high as 29% (1, 13, 14). However, rates of suicidal ideation amongst youth are likely to be even higher than estimated, as young people are often reluctant to disclose this (1).

With high rates of suicidality amongst young people, it is important to understand the dynamics of this experience in order to identify needs and opportunities for intervention. Some research documents the broad patterns of suicidality, suggesting that at the start of adolescence, the risk of the first onset for suicidality significantly increases, peaking at age 16, and remains elevated until the young person's early twenties (14, 15). However, findings from longitudinal trajectory studies suggest that, within this, there is immense heterogeneity among young people experiencing suicidality and that different courses of suicidality exist (7, 16–19). Despite differences in trajectories, there is a general consensus among researchers that suicidality among young people is thought to be dynamic, with some studies suggesting that an escalation often occurs over time (14, 15).

Variability in suicidality over short time periods has also been documented among young people. Using mobile phone technology, Czyz and colleagues captured daily records of young people's suicidality in the month following a suicide attempt and found considerable day-to-day fluctuations in the frequency, duration, and urge severity of suicidal ideation (20). Although this study offered a fine-grained account of young people's experience of suicidality, the study was limited as young people had to choose their responses from predetermined answers, which would have reduced the opportunity to explore less well-recognized patterns of suicidality.

Many studies suggest that suicidality among youth is often a recurring experience (21), with research indicating that if a young person experiences suicidality, they are at increased risk of future suicidal ideation, attempts, and suicide (12, 22–24). In line with this, a number of studies suggest that the experience of suicide among young people can be pervasive and ongoing (7, 25, 26). Despite this, the escalation from ideation to action appears to occur very rapidly (27). For example, one study showed that only 21% of young people who attempted suicide had planned their suicide attempt for more than 24 h in advance (26). As impulsivity has been associated with an increased risk of suicidality among young people, it is clearly important to understand more about the period immediately prior to the enactment of suicidal behavior (28).

Although growing attention has been given to the course of suicidality, there is still limited understanding of young people's own experience of feeling suicidal (29). Young people's own accounts of suicidality have the potential to add depth and nuance to the existing knowledge about the dynamics of the phenomenon. A relatively small number of qualitative studies have explored young people's own experiences of suicidality through interviews (25, 30–32). This research has drawn attention to the emotional state of young people experiencing suicidality, highlighting their experience of despair, shame, and social disconnection (25). There is also research that suggests that there may be important shifts in motivation and intentionality within episodes of suicidality (30–34). An enhanced understanding of the emotional states and motivations of young people experiencing suicidality might open up opportunities for more targeted intervention during crisis periods.

Due to the stigma associated with suicide, ethical concerns, and young people's reluctance to seek support for their difficulties, only a few studies provide direct access to “in the moment” experiences of suicidality. Although some recent research on internet forums has captured more direct expressions of suicidality in the moment they are felt, the data is often limited by the constraints of the platform on which the young people are communicating (35–38). In order to refine suicide prevention in this age group, it is vital to understand young people's own experiences of suicidality (17, 29). This article seeks to address gaps in the literature by exploring young people's real-time communications about their suicidality during a period of crisis.

## Materials and Methods

This article draws from a qualitative analysis of young clients' communications about their suicidality, provided in the context of a text counseling interaction with a helpline service. The service was run by a youth development agency, Youthline, which provides a free interactive text message counseling service for youth 12–24 years of age experiencing distress. Youthline's text counseling service operates between 8 am and midnight, seven days a week. The text message service can be accessed by any young person in New Zealand, provided they have mobile phone reception. It is an anonymous service that does not require young people to sign up, register, or provide personal information (39). The text counseling sessions consist of asynchronous exchanges between a trained volunteer counselor and a client, which are automatically recorded and stored in text format. While clients text the service with a range of issues, suicidality is a major theme in the content of these counseling sessions and a significant concern for the organization. Counselors generally aim to prevent suicide by talking the young client through their crisis and referring them for ongoing mental health support if required. While clients are anonymous to the service, the police can be called in to locate a client when there are significant concerns about their safety.

For the purposes of this study, we analyzed text counseling transcripts to identify common patterns in the clients' communications about their suicidality, including the reasons they provided for feeling suicidal, where they had sought help, and why. This article, however, addresses the more focused question: “How do those clients who say they are suicidal, describe their experience of this phenomenon?”

A social-constructionist epistemology informed our research with an awareness that suicide is always constructed by someone for a particular purpose and in some context (40). Our position was also influenced by a youth empowerment perspective, which recognizes the importance of understanding the meaning that young people themselves give to their experiences (41). This approach requires a reflexive awareness of the researcher's own positioning in relation to the research participants and the analysis (42). The first researcher was a clinical psychology doctoral student who was working in a community mental health setting at the time of the study. The second researcher was a clinical psychologist and academic who served as the supervisor on the project. Both researchers were particularly conscious of needing to challenge their professional preconceptions to allow the experience of the young clients to be heard more clearly.

### Data Collection

The data consisted of text message counseling transcripts which Youthline stored as part of their usual clinical and audit practices. The criteria for a text conversation to be included in the analysis was that the client communicated experiencing suicidality while messaging the service. A Youthline staff member searched for the word “suicide” in their database on 7 February 2017 and collected 200 of the most recent text message transcripts.

The researchers then reviewed the 200 transcripts to ensure they included references to current experiences of suicidality, including suicidal ideation, suicide planning and suicide-related behaviors. To distinguish between non-suicidal self-injury and suicidality, only text interactions where the client communicated explicit references to wanting to die or end their life were included. Of the 200 conversations collected by Youthline, five were excluded as they were repeats, and 70 were excluded as they did not meet the study criteria. The 70 excluded transcripts included: one conversation where the client texted that they were not a young person; 17 transcripts where the client was communicating concerns for someone else who was experiencing suicidality; 42 transcripts where the client was distressed but was not experiencing any current suicidality; and ten conversations where the client's experience of suicidality was unclear, and the counselor did not ask about the client's current suicidality.

This resulted in 125 text message counseling transcripts where the client communicated experiencing current suicidality. The data contained all text communication between the counselor and the client. In total, 5,933 text messages were included in the analysis. The text message interactions ranged between 4 and 184 text messages, with the average text conversation consisting of 47 messages. Saturation, the point at which additional data does not appear to generate new themes, is commonly used to determine sample size in qualitative research (43). In this study, saturation was judged to have been reached with the 125 transcripts available, with no need to request further transcripts from Youthline (43).

### Data Analysis

In order to locate recurring themes in the data, the researchers employed thematic analysis. This form of analysis was appropriate for this study as it is thought to be useful when exploring an under-researched area. Furthermore, as it allows for the latent aspects of the data to be identified by going beyond the semantic content of the texts, it was consistent with a social-constructionist epistemological position (42, 44). While the research question offered a foundation to guide the interpretation of the data, specific themes were not predetermined and instead were identified during the analytic process. This method of analysis allowed us to identify the themes associated with our research question whilst also permitting what the young people communicated to inform new findings (44).

The data was analyzed using Braun and Clarke's approach to thematic analysis (44). This provided a flexible method for identifying, analyzing, and interpreting themes within the transcripts. Throughout the analysis, the researchers discussed and reviewed the themes to ensure trustworthiness, using consensus and reflexive discussion as the basis for our decisions (42, 45).

Following Braun and Clarke's guidelines, the first step in the analysis was becoming familiar with the content by reading and re-reading the text message conversations multiple times (42). This allowed us to clarify the research question addressed in this article. Once we had identified this research question, we went through all the data and extracted any communications that related directly to the clients' experiences of suicidality. This research question necessitated that we focus specifically on the clients' communications (using the counsellor's questions and responses only as context for locating or understanding the meaning of the clients' communications).

The analysis began with multiple readings of the transcripts, which helped us to generate initial codes to organize the data into meaningful categories. Each transcript was coded with as many codes as were relevant. Extracts and quotes from the conversations were recorded under each code to support the elucidation of content and meaning. In order to keep true to the clients' communicated experience, minimal changes were made to grammar and misspellings, and “text speak” was not altered. Once all the data had been coded, the preliminary themes were identified, with the relevant data placed under each theme. The research team then reviewed the themes to ensure the themes were well supported, captured the range of clients' experiences, and sufficiently well differentiated from one another. While this is described as a linear process, as Braun and Clarke note, analysis often involves an iterative process of moving backwards and forwards between the data and high levels of analysis (44).

We were conscious of ethical considerations in researching sensitive issues such as suicide. Youthline consented to the study, and we worked closely with the counseling team to ensure clients were protected. As clients were anonymous to the organization it was not appropriate or feasible to gain client consent to use their counseling transcripts in this study. While there are ethical issues related to using these transcripts without the clients' permission, these considerations were carefully weighed against other imperatives including the value of improving the suicide crisis service for young people and the discomfort clients might experience if they were approached via their mobile phone numbers to request retrospective consent. Analyzing anonymised file data, previously collected in the course of usual practice, is common practice in health organizations, and is considered part of an obligation to evaluate and improve their services. Consistent with these audit practices, all data was carefully anonymised before being provided to the researchers. Furthermore, in writing up the research, we have avoided highlighting idiosyncratic responses or the details of any specific situation described by a client to protect client privacy further. The research was approved by the University of Auckland Human Participants Ethics Committee.

## Findings

Through the analysis, we identified eight themes that reflected clients' experiences of suicidality.

### A Normal Part of Life: “Thoughts Are Becoming Part of an Everyday Thing Now”

Many clients described experiencing persistent and ongoing suicidal thoughts marked by an ongoing feeling of hopelessness. This theme illustrated how suicidality was communicated as a chronic and pervasive experience that had become a re-occurring and normal part of their lives.

Clients texted that they thought about suicide all the time. They frequently communicated experiencing suicidality for days, weeks, and even years. As one client messaged, “I'm always thinking about it these days” (Client 109). Clients talked about their suicidality as being a pervasive experience, describing their suicidal thoughts as “really loud” (Client 117), “really strong” (Client 68), and “oppressive” (Client 85). Due to the chronic nature of their suicidality, clients communicated feeling like they were in a constant struggle. For example, one client messaged, “Every time I close my eyes for more than like 5 min I imagine myself dying” (Client 59). They messaged that they had little hope of no longer experiencing suicidality. As one client texted, “They've calmed down but there's always going to be that thought in my mind” (Client 67).

Clients also messaged that their experience of suicidality was recurring. As one client texted, “this is a very common occurrence for me” (Client 115). They noted a range of different experiences of suicidality in the past, such as suicidal ideation, suicide attempts, and sometimes periods in respite and hospitalization. These clients often reported having attempted suicide multiple times. For some of these clients, these attempts were very recent, such as the day before they messaged the service.

These clients communicated that as their experience of suicidality was so frequent and persistent, suicide had become a normal part of their life. As one client texted, “Thoughts are becoming part of an everyday thing now” (Client 104). For many, their experience of suicidality had become part of how they saw themselves; as one client texted, “I want to kill myself. Which is kind of normal for me” (Client 25). They often conveyed that their experience of suicidality had become part of their identity. For example, when asked about the frequency of their suicidal thoughts, one client replied, “Most of the time. Nobody is even surprised anymore more cause I have ‘chronic' suicidal ideation” (Client 117).

### A Form of Coping: “Death Is a Better Option”

A number of clients described suicide as being an understandable response given what they were experiencing and a logical way to solve their problems. This theme captured how suicidality was often understood as a way of coping.

The clients communicated seeing suicide as being a reasonable response given their emotional pain. Suicide was seen as a way to relieve their distress and escape from their painful emotions. This is shown in the interaction below.

Client 39: I'm just really fed up and exhausted of trying to get better all the time.
Youthline 39: Getting better can be a slow process and understandable that you're feeling discouraged.
Client 39: Yeah, it just seems like death is a better option.
Youthline 39: Do you think death is a better option or do you want the pain to stop, just trying to understand where you're at.
Client 39: Both. I want the pain to stop and I think death is the best chance of that happening.

Clients often did not speak of the finality of taking their life and instead described it as a way to gain “instant relief” (Client 68) and peace from their strong and unwanted emotions. Clients also communicated that suicide would solve their problems, often conveying the idea that it was a logical and reasonable option. For example, one client texted, “the best way for me get out of this is kill myself” (Client 83). They conceptualized suicide as being a coping strategy in itself, which was viewed as accessible and understandable. For instance, when asked by their text message counselor what coping strategies they had used in the past, one client gave attempting suicide as an answer (Client 87). Furthermore, clients frequently described suicide as being their only option, as this client texted, “It seems like the only way to escape everything” (Client 117).

### Ambivalence About Suicide: “Part of Me Wants to and Part of Me Doesn't”

Many clients conveyed feeling ambivalent about taking their life. This theme highlighted the uncertainty and internal life-or-death debate that clients frequently communicated in their texts.

Clients often talked about feeling ambivalent about taking their life and texted that despite not wanting to be dead, suicide was always an option. For example, one client messaged, “There's obviously a part of me that doesn't want to die but all my other problems just seem to overrule the small part of me that wants to live” (Client 117). A number described experiencing a constant internal conflict, where they were unsure or did not want to take their life but felt unable to cope with their painful emotions or difficult situation. For example, one client messaged, “I don't really want to commit suicide, I just want the pain to end (Client 57). Clients frequently said that it was not that they wanted to be dead, but they wanted to solve their problems. As this client messaged, “I want to leave this world. But I don't want to die” (Client 112). They described being afraid of taking their life and did not want to do it but could see no other option. For instance, one client texted, “I'm scared to do it. I know deep down I don't want to but I can't see any other way out” (Client 89). Clients communicated being confused and uncertain about taking their life and frequently texted that they did “not know what to do”. For example, one client messaged, “I don't know what to do because I just want everything to end” (Client 113).

Clients also communicated ambivalence regarding having a plan to take their life and what means they would use. They often used words such as “maybe”, “not yet”, and “probably” when answering questions surrounding intent. For example, in response to being asked about the likelihood of them acting on their suicidal thoughts, one client texted, “Part of me wants to and part of me doesn't at the moment I'm just sitting at a riverbank thinking. It's just a difficult decision but I just feel really down” (Client 83).

### A Way to Communicate Distress: “I Just Don't Have Anyone I Can Talk to”

For many clients, their suicidality was a way of communicating their distress and pain. This theme discussed how clients often used suicidality to convey their anguish and connect with others.

Clients repeatedly said that suicidality was a way to express their pain. For example, one client texted, “Sometimes I have outbursts where I cry and say stuff but I never actually have intentions of doing it you know?” (Client 73). Often, when a client's desire to take their life was explored further by their counselor, it was found that their suicidality was a way to express their distress, and they did not want to die. This is highlighted in the interaction below.

Client 78: I wanna die.
Youthline 78: Hey hearing that you want to die. Just wanting to check your safety do you currently have a plan to end your life?
Client 78: No I just sick of being me and hurting.
These clients appeared to express suicidality as a way to receive support from others. An example of this is shown in the text message interaction below.
Client 14: I really want to die, I have no reasons to live anymore.
Youthline 14: We r concerned for u Are u intending to suicide tonight?
Client 14: No, I just don't have anyone I can talk to.

### Increasing Intensity: “It's Getting Worse”

A number of clients communicated that although they always experienced some kind of suicidal ideation, the intensity of their suicidality varied over time. This theme highlighted how clients described their experience of suicidality as dynamic, with clients noting a gradual worsening and rapid shift in intensity over short time periods.

These clients communicated that their suicidal ideation was increasingly getting “worse” and “stronger”. They frequently texted that their suicidal thoughts continued to increase in frequency and intensity over days and weeks. This is highlighted in the text interaction below.

Client 100: I just don't really know what to do.... it's getting worse.
Youthline 100: When you say it's getting worse what do you mean?
Client 100: Like the whole suicide thing.. it's on my mind more and more each day.

For many, as their suicidal ideation increased in intensity, so too did their behaviors that could increase their risk to themselves, such as not taking their medication and planning how they would take their life. For example, one client messaged, “Well ever since feeling like killing myself last Friday I've just felt odd and it's kind of been getting worse and worse every day which is why I don't feel like taking my antidepressants” (Client 47).

While most clients noted that their suicidality as a whole was worsening over time, clients also described the intensity of their suicidality increasing and decreasing over a short space of time, such as over hours or days. This is highlighted in the quotes below.

Client 60: My thoughts are very violent.
Youthline 60: What sort of violent thoughts are you having, just concerned for your safety.
Client 60: I don't know they've gone back to being murmurs in my head.

It was often unclear what exacerbated or alleviated these clients' distress. At times, they communicated increased suicidality in response to arguments with parents and friends and when they were experiencing strong emotions, such as anger and sadness. While other clients noted their suicidality increased when they were by themselves, particularly at night. For example, one client messaged, “It usually happens at night. When I'm alone it's just being alone makes me feel worse because that's when my mind goes crazy” (Client 100).

### Out of Control: “My Head Takes Over”

A number of clients described their experience of suicidality as being out of their control. This theme explored how clients communicated having limited control over their experience of suicidality, highlighting the helplessness many described experiencing.

These clients conveyed a sense of their suicidality as being an external force that had consumed them. This is shown in the interaction below.

Client 7: My head takes over and I can't get away from it.
Youthline 7: Can u tell us more about what u mean about your head taking over?”
Client 7: I try distract myself but my thoughts just win. They always come back so much worse and they get so unbearable to the stage where I'm hurting myself, taking pills, drinking and more.

Clients frequently described having no control over this force, often conveying a sense of helplessness. As one client texted, “I can't make it stop” (Client 59). They communicated feeling like they had very little or no control over their risk of attempting suicide. For example, one client messaged, “I'm scared because I don't know what's going to happen next or whether suicide will get to me first before the help starts working mainly” (Client 91). Clients repeatedly texted that they were unsure if they could keep themselves safe. They also had difficulty answering questions regarding the likelihood of them acting on their suicidal thoughts and often responded to questions regarding intent with “I do not know”. For instance, one client messaged, “No one knows...one day I might actually go too far with my attempts or self-harm. Even I don't know. Till the time comes” (Client 19). They also communicated being frightened that they would take their life. As this client texted, “I'm scared of what i might do to myself” (Client 72).

Clients also often described experiencing a constant battle with their experience of suicide. For example, one client messaged, “It is very hard: (I don't know how much longer I can fight:)” (Client 50). They texted saying they felt “tired” and described feeling defeated with the constant struggle with their suicidality. As this one client texted, “I've given up trying and I'm already gone” (Client 37).

### Planning Suicide: “I Have Made a Plan on How. Several Plans. In Case Others Fail”

Around half of the clients communicated having thought of how they would suicide, while the other half described experiencing suicidal thoughts with no plan or intent to act on their thoughts. This theme discussed the different levels of suicidal intent communicated by clients, highlighting that many had thought about how they would suicide, some were in the process of taking their life, and the remaining clients were experiencing suicidal thoughts with no intent.

The analysis suggested that around half of the clients communicated having thought about how they would suicide. These clients frequently talked about having very specific and detailed plans to end their life. They described having put a lot of thought into how they would take their life and reported thinking about their plan for a long time. Clients discussed leaving suicide notes for their loved ones and described researching ways to kill themselves online. For example, one client texted,

“Um so it would probably be about 1 or 2 in the morning and a Saturday and I'll be at my uncles. I'd have a note for my parents when they come to pick me up to tell them why. But I'd probably hang myself in the shed with a belt” (Client 101).

Often, clients noted multiple ways they could suicide and consequently had more than one suicide plan. For example, one client messaged, “I have made a plan on how. Several plans. In case others fail. It'll just happen when the time and day is right” (Client 23). These plans involved jumping off buildings or bridges, hanging, overdosing on a range of substances, cutting, and running into traffic. For instance, one client texted, “Well my school is in town so I figured at break I'd wonder off find some rope and hang myself. Or jump off a building” (Client 118).

At times, clients said they had started preparing and carrying out their suicide plan, such as stockpiling medication. For instance, one client messaged, “I have taken like 5 anti-depressants from my brother every month for the past year and a bit. They are in my drawers” (Client 113). A small number messaged that they planned to take their life right then. For example, one client texted, “Yip before I go to bed I am going to use all my sister's sleeping pills and hope I don't wake up in the morning” (Client 110). Some clients were already attempting suicide before or while messaging in to Youthline. This is shown in the interaction below.

Client 119: Im hurting myself!!
Youthline 119: What do you mean?
Client 119: Im cutting and i feel like it.
Youthline 119: Is that really going to help?
Client 119: Yep cut deeper and deeper.

The majority of these clients said they did not want other services to get involved, such as emergency services or a mental health crisis team. This is highlighted in the interaction below.

Youthline 52: We are really concerned for ur safety right now. Can u please text us with ur address?
Client 52: Why do you want my address?
Youthline 52: We are concerned about u. U have said u have cut urself, taken pills and alcohol & we want to help u by getting u support right now. We can do this together. If u need the ambulance, we can call them. If we know where u r we can contact ur local crisis team to help.
Client 52: No. I just want to die.

These clients often declined Youthline's offer to call them and did not answer when their text message counselor tried to call them. However, at times they did eventually engage with supports, such as the mental health crisis team or emergency services or agreed to speak with Youthline over the phone. For instance, in the above example (Client 52), the client gave their contact details and Youthline was able to connect them with further support.

Interestingly, clients very infrequently described their suicidality as being impulsive. However, a small number of clients implied that their suicidal plans were impulsive and messaged that they planned on using whatever means they could find first or whatever was most accessible. For example, one client texted, “I'm alone and on the streets upset and I don't care how I'm just going to find a way to jump in front of a speeding truck, train, buildings, bridge, cliff anyway I find first” (Client 56). For those few who described their suicidal plans in this way, it often seemed in response to interpersonal difficulties, such as a fight with a family member or a peer. Of note, these clients often still described experiencing ongoing and persistent suicidality that preceded their current crisis.

The remaining half of the transcripts suggested that clients experienced suicidality with no intent to act on their thoughts. These clients communicated experiencing frequent suicidal thoughts but had not thought about how they would take their life and had no obvious plan to kill themselves. As one client texted, “I'm not suicidal. Passively wanting to die and being actively suicidal are different things” (Client 32). This theme highlighted the heterogeneity among clients in regards to their suicidal intent.

### Recognizing Help Was Needed: “I Need to Get Help”

A number of clients acknowledged needing help and support from others for their suicidality. This theme discussed how clients recognized that they needed help for their suicidality and what help they wanted.

These clients acknowledged they needed support from family or friends and sometimes more formal supports, such as a mental health crisis team, respite services, and school counselors. For example, one client texted, “I actually want to kms [kill myself], it's just idk [I don't know] I hate living I need to get help:/” (Client 22). However, at times, clients said they did not want support from others, which included talking to family, friends, or professionals. For instance, one client messaged, “No i DONT WANT HELP (Client 56). Clients also oscillated between wanting and receiving support from the service. These clients refused to answer their counsellors' questions or responded in a manner that was challenging. This is reflected in the interaction below.

Youthline 28: Do u have a plan 2 end ur life?
Client 28: Yeah.
Youthline 28: Can you please share more about your plan?
Client 28: No…You don't need to know.

Despite sometimes expressing reluctance about having a service intervene in their suicidal plans, clients repeatedly communicated wanting “someone to talk to” and support people who would listen to them. As one client texted, “My suicidal thoughts are back and I need help and no one's listening to me” (Client 48). They described wanting their support people to “care", trying to understand their feelings without judgment, and not minimizing their experience. For example, one client messaged, “I just need someone who understands me and would let me open up” (Client 22). Clients often said they did not want to be told what to do and instead wanted space to talk and be heard. As one client texted, “I know people care but I feel like I just need a friend. Who doesn't try and fix me or doesn't get it, but is just there” (Client 93).

## Discussion

This research offers unique insights into young people's experiences of suicidality in the moment of reaching out for help. The findings of this research counter the perception that young people are unwilling to ask for help with suicidality and instead suggests that some are desperately seeking timely help to manage and overcome their suicidality. The difficulty of engaging suicidal youth with help has often been attributed to their developmental priorities and concerns including an investment in autonomy and mistrust of authority (6–9). It might however be that many young people do in fact want help, but that services themselves are not well set up to respond to young people's distress in a respectful, timely, and developmentally appropriate manner (9, 46).

The findings of this research also challenge the idea that youth suicidality is necessarily unpredictable and impulsive and instead suggests that some young people are experiencing an ongoing struggle with suicidality. Although the young people who texted the helpline were clearly in a state of crisis, suicidality was frequently perceived as a persistent and ongoing experience, which was described as a constant and exhausting struggle (7, 26). Our findings highlight the importance of recognizing patterns of chronic suicidality amongst young people This underlines an urgent need for accessible primary mental health services specifically designed to be acceptable to youth (46).

As these young people's experience of suicidality was so frequent and chronic, suicide was often communicated as being a normal part of their life and seemed to have been integrated into their sense of self. These young people's individual struggles with suicidality might be reinforced by representations of suicidality as ubiquitous and normative, as has been suggested by researchers who have considered this phenomenon in contemporary youth cultures (47–49). The potential normalization of suicide amongst some young people highlights the need for a delicate balance of reducing understandings of suicide as a viable option whilst simultaneously decreasing the stigma associated with talking openly about suicide (48).

Interestingly, young people did not speak about the finality of ending their life and instead conveyed a perception that suicide would bring them peace and salvation. For many young people in this study, their motive was not solely to die, and other functions of their suicidality appeared to be in wanting to solve their problems or escape from emotional pain. It seemed that young people viewed suicidality as being a coping strategy in itself. Suicidal ideation and behaviors were often conveyed as being the only way these young people could cope with their difficulties and solve their problems (50–52). Our study also captured the lack of control that young people felt in dealing with the experience of suicidality itself. This is in line with the findings of Lachal and colleagues' meta-synthesis of qualitative studies, which suggested that young people experience a loss of self-control during episodes of suicidality (52). This highlights the value of interventions that might improve young people's sense of mastery, problem-solving ability, and emotion regulation skills (25, 53).

The young clients in our study also conveyed that suicide was a way for them to communicate pain and distress (32, 54). This is in line with research, which suggests that suicidality among young people might serve an interpersonal function (31). Some researchers have argued that revenge was an aspect of this communication (29). However, our findings did not support this and instead indicated that the motivation behind suicidal young people's behavior was to alleviate and share their suffering (55, 56). Some researchers have argued that young people might lack the language to convey emotional pain effectively (25, 33). However, it may also be that young people are not given opportunities and encouragement to express their unhappiness. This may be especially pertinent in New Zealand, where young people feel their expressions of distress are silenced by those around them, including their families and broader society (46, 57). This underlines the importance of opening communication channels for young people to convey their distress prior to the point at which they are in crisis.

One of the most practically pertinent findings of this research was that suicidal youth often experience ambivalence surrounding taking their life. In our study, the clients who contacted the helpline were largely uncertain about whether they wanted to kill themselves. This ambivalence suggests a unique opportunity for counselors to ally with the part of the client that wishes to live. Similar ambivalent intent has been demonstrated among young people who attempt suicide and requires further research and clinical attention (12, 30, 32). Services that operate 24-h a day may be particularly important for young people experiencing suicidality, so that they have access to support in their moment of indecision.

Our analysis also underlined the heterogeneity and dynamic experience of suicide both within and among young people. Many of the text messages suggested that the intensity of young people's suicidality gradually worsened over time (14, 15). However, within this steady worsening, clients also appeared to experience intense fluctuations in their suicidality over very short periods of time. This is congruent with previous research, which suggests that suicidality may be dynamic and changing (20). This finding supports the need to take the possibility of rapid fluctuations of suicidality into account when designing and carrying out risk assessments with young clients.

While not all of the clients whose transcripts were analyzed in this study communicated having a suicide plan, many did communicate that they had clear plans of how they would take their life and had considered a method of suicide. These methods were often accessible, such as overdosing on over the counter medication and hanging themselves. This highlights the importance of suicide prevention initiatives, including suicide safety planning, that targets restricting access to suicide means where possible (57, 58). The suicide plans the young people described were often also highly detailed, some indicating that multiple options for carrying this out had been considered. This suggests that suicidal young people may spend prolonged periods thinking about ways they could take their life. This supports other research noting that young people's experience of suicidality may precede any noticeable “at risk” behaviors (7, 26). Although these findings contrast with many of the dominant understandings of youth suicide as being impulsive, they are congruent with studies that have found premeditation is a stronger predictor of suicidality than impulsivity (15, 59). However, this finding may also reflect a particular subsection of the youth population who can avert a suicidal impulse long enough to reach out for help.

Although less common, some young people in the study also texted an immediate intent to take their life and noted carrying out suicidal behaviors before and while messaging the service. This suggests that, although some youth may be ambivalent about suicide, others experiencing suicidality do experience imminent intent and attempt suicide (1, 60). Although these young people frequently communicated not wanting emergency support, it is important to emphasize that they did message in for help. This indicates that young people experiencing suicidality may want support at their critical or heightened moment of distress, but it may be a specific kind of support responsive to their individual needs and wants (61).

Importantly, our analysis suggests that young people experiencing suicidality often acknowledged that they needed help, indicating they had some insight into their difficulties and contrasts with previous research, which found suicidal youth do not recognize the need for support (6, 8). Previous research indicates that young people are more willing to engage with text counseling support in a crisis than with telephone or face-to-face counseling. This underlines the importance of making accessible helplines available to young people who are experiencing suicidality (62). Further research is urgently needed to establish whether the advantages of accessibility and acceptability of text counseling for young people are matched with their effectiveness in preventing suicide.

### Limitations

Due to the anonymity allowed by the helpline service, the clients' data was unknown. Consequently, important information, such as the client's age, gender, and ethnicity remained undetermined. However, this research aimed not to provide a statistical generalization to specific populations but to gain an in-depth understanding of young people's communication of their experience of suicidality. Further limitations of the data related to the context of the counseling session. While, in some cases, the counsellors' questioning was able to elicit further information, it was also constrained by the counsellors' responses and the format and purpose of the counseling situation.

## Conclusions

There is limited research in the youth suicide literature from the perspectives of young people themselves. This study provided further understanding of suicidality among young people by focusing on how they themselves communicated their experience of the phenomena in real-time. The insights they provide into the experience of suicidality have important implications for both prevention and early intervention strategies in youth suicide.

## Data Availability Statement

The datasets presented in this article are not readily available because the data consists of sensitive material collected during a crisis helpline interaction. We only have ethics permission for the authors access to these. Requests to access the datasets should be directed to kl.gibson@auckland.ac.nz.

## Ethics Statement

The studies involving human participants were reviewed and approved by University of Auckland Human Participants Ethics Committee. The Ethics Committee waived the requirement of written informed consent for participation.

## Author Contributions

JV was the first author and carried out the research. KG supervised the research, assisted with the data analysis, and write up. All authors contributed to the article and approved the submitted version.

## Conflict of Interest

The authors declare that the research was conducted in the absence of any commercial or financial relationships that could be construed as a potential conflict of interest.

## Publisher's Note

All claims expressed in this article are solely those of the authors and do not necessarily represent those of their affiliated organizations, or those of the publisher, the editors and the reviewers. Any product that may be evaluated in this article, or claim that may be made by its manufacturer, is not guaranteed or endorsed by the publisher.
